# Mid-life epigenetic age, neuroimaging brain age, and cognitive function: coronary artery risk development in young adults (CARDIA) study

**DOI:** 10.18632/aging.203918

**Published:** 2022-02-27

**Authors:** Yinan Zheng, Mohamad Habes, Mitzi Gonzales, Raymond Pomponio, Ilya Nasrallah, Sadiya Khan, Douglas E. Vaughan, Christos Davatzikos, Sudha Seshadri, Lenore Launer, Farzaneh Sorond, Sanaz Sedaghat, Derek Wainwright, Andrea Baccarelli, Stephen Sidney, Nick Bryan, Philip Greenland, Donald Lloyd-Jones, Kristine Yaffe, Lifang Hou

**Affiliations:** 1 Department of Preventive Medicine, Northwestern University Feinberg School of Medicine, Chicago, IL 60611, USA; 2 Biggs Institute Neuroimaging Core, Glenn Biggs Institute for Neurodegenerative Disorders, University of Texas Health Science Center at San Antonio, San Antonio, TX 78229, USA; 3 Department of Radiology, University of Pennsylvania, Philadelphia, PA 19104, USA; 4 Division of Cardiology, Department of Medicine, Northwestern University Feinberg School of Medicine, Chicago, IL 60611, USA; 5 Feinberg Cardiovascular Research Institute, Northwestern University Feinberg School of Medicine, Chicago, IL 60611, USA; 6 Department of Neurology, Boston University School of Medicine, Boston, MA 02118, USA; 7 Laboratory of Epidemiology and Population Science, Intramural Research Program, National Institute on Aging, National Institutes of Health, Bethesda, MD 20892, USA; 8 Department of Neurology, Northwestern University Feinberg School of Medicine, Chicago, IL 60611, USA; 9 Division of Epidemiology and Community Health, School of Public Health, University of Minnesota, Minneapolis, MN 55455, USA; 10 Departments of Neurological Surgery, Medicine-Hematology and Oncology, Microbiology-Immunology, Northwestern University Feinberg School of Medicine, Chicago, IL 60611, USA; 11 Department of Environmental Health Sciences, Columbia University Mailman School of Public Health, New York, NY 10032, USA; 12 Kaiser Permanente Division of Research, Oakland, CA 94612, USA; 13 Department of Diagnostic Medicine, Dell Medical School, University of Texas at Austin, Austin, TX 78712, USA; 14 Departments of Psychiatry and Behavioral Sciences, University of California, San Francisco, CA 94143, USA; 15 Department of Neurology University of California, San Francisco, CA 94143, USA; 16 Department of Epidemiology and Biostatistics, University of California San Francisco, CA 94143, USA; 17 San Francisco VA Medical Center, San Francisco, CA 94143, USA

**Keywords:** cognitive function, epigenetic age, brain age, DNA methylation, magnetic resonance imaging

## Abstract

The proportion of aging populations affected by dementia is increasing. There is an urgent need to identify biological aging markers in mid-life before symptoms of age-related dementia present for early intervention to delay the cognitive decline and the onset of dementia. In this cohort study involving 1,676 healthy participants (mean age 40) with up to 15 years of follow up, we evaluated the associations between cognitive function and two classes of novel biological aging markers: blood-based epigenetic aging and neuroimaging-based brain aging. Both accelerated epigenetic aging and brain aging were prospectively associated with worse cognitive outcomes. Specifically, every year faster epigenetic or brain aging was on average associated with 0.19-0.28 higher (worse) Stroop score, 0.04-0.05 lower (worse) RAVLT score, and 0.23-0.45 lower (worse) DSST (all false-discovery-rate-adjusted p <0.05). While epigenetic aging is a more stable biomarker with strong long-term predictive performance for cognitive function, brain aging biomarker may change more dynamically in temporal association with cognitive decline. The combined model using epigenetic and brain aging markers achieved the highest accuracy (AUC: 0.68, p<0.001) in predicting global cognitive function status. Accelerated epigenetic age and brain age at midlife may aid timely identification of individuals at risk for accelerated cognitive decline and promote the development of interventions to preserve optimal functioning across the lifespan.

## INTRODUCTION

By the year 2030, 75 to 82 million people worldwide are projected to be affected by dementia [1, 2]. Early diagnosis is valuable for timely management and intervention to delay or prevent cognitive decline and the onset of dementia [3–5]. Cognitive abilities decline with advancing age [6]. However, despite cumulative downward trajectories of cognition, prior studies have shown marked heterogeneity in the rate of decline across individuals [7–11]. This highlights the need for new approaches for the early detection of cognitive decline, based on biomarkers of systematic age-related biological degeneration including molecular aging markers in blood, as well as the degeneration directly in brain captured by structural brain imaging [12]. As both of these markers have high potential to inform and predict future cognitive status at the individual level, identifying biological blood- and imaging-based aging markers associated with cognitive function in mid-life, decades before symptoms of age-related dementia present, may aid in early detection of possible disease in people with mild symptoms, and facilitate the identification of vulnerable individuals before the onset of irreversible neuronal damage and extend opportunities for intervention.

Extensive basic science and epidemiological research have indicated that age-related cognitive decline is governed by interactions across genetic and environmental factors [13–15]. Epigenetics is a molecular marking system that reflects environmental and lifestyle factors [16]. DNA methylation (DNAm) is one of the most well-established epigenetic mechanisms linked to aging and aging-related diseases, [17] and it can be used to assess biological aging. The multi-tissue-derived Horvath's DNAm age and blood-derived Hannum's DNAm age are predictive of chronological age [18, 19]. More recently, newer blood-derived epigenetic aging models, such as DNAm Phenotypic Age (PhenoAge) [20] and GrimAge [21] have been developed that derive their DNAm predictions of life expectancy and risk of mortality from markers of physiological dysregulation. In particular, the latest GrimAge model can inform incident cardiovascular disease and all-cause mortality with stronger and more significant associations than earlier DNAm age measures [21].

While blood-derived epigenetic aging markers have shown predictive value years before age-related diseases occur [21–23]. biological aging rates can differ across organ systems, so predictors derived directly from the brain may hold unique information for cognition [24, 25]. Across the lifespan, aging-related brain atrophy occurs in a predictable manner [26, 27]. Leveraging machine-learning algorithms, a composite age-related morphological index, Spatial Pattern of Atrophy for Recognition (SPARE) of Brain Age (SPARE-BA), has been developed to translate atrophy of brain structures into an aging marker [28–30]. As compared with resilient older adults, individuals with advanced imaging brain age have been found to display worse verbal fluency and attentional skills [10].

To study the portion of biological age that is not explained by chronological age, the concept of “age acceleration” has been proposed, [19] which quantifies the independent deviations of the biological age from chronological age. A positive value of age acceleration indicates that the biological age is higher than expected based on chronological age. The goal of the present study was to quantify the associations of epigenetic age acceleration and SPARE-BA acceleration with subsequent cognitive performance in a biracial cohort (~40% Black participants and ~60% White participants) of middle-aged adults with 5 to 15 years of follow up.

## RESULTS

**Figure 1:**
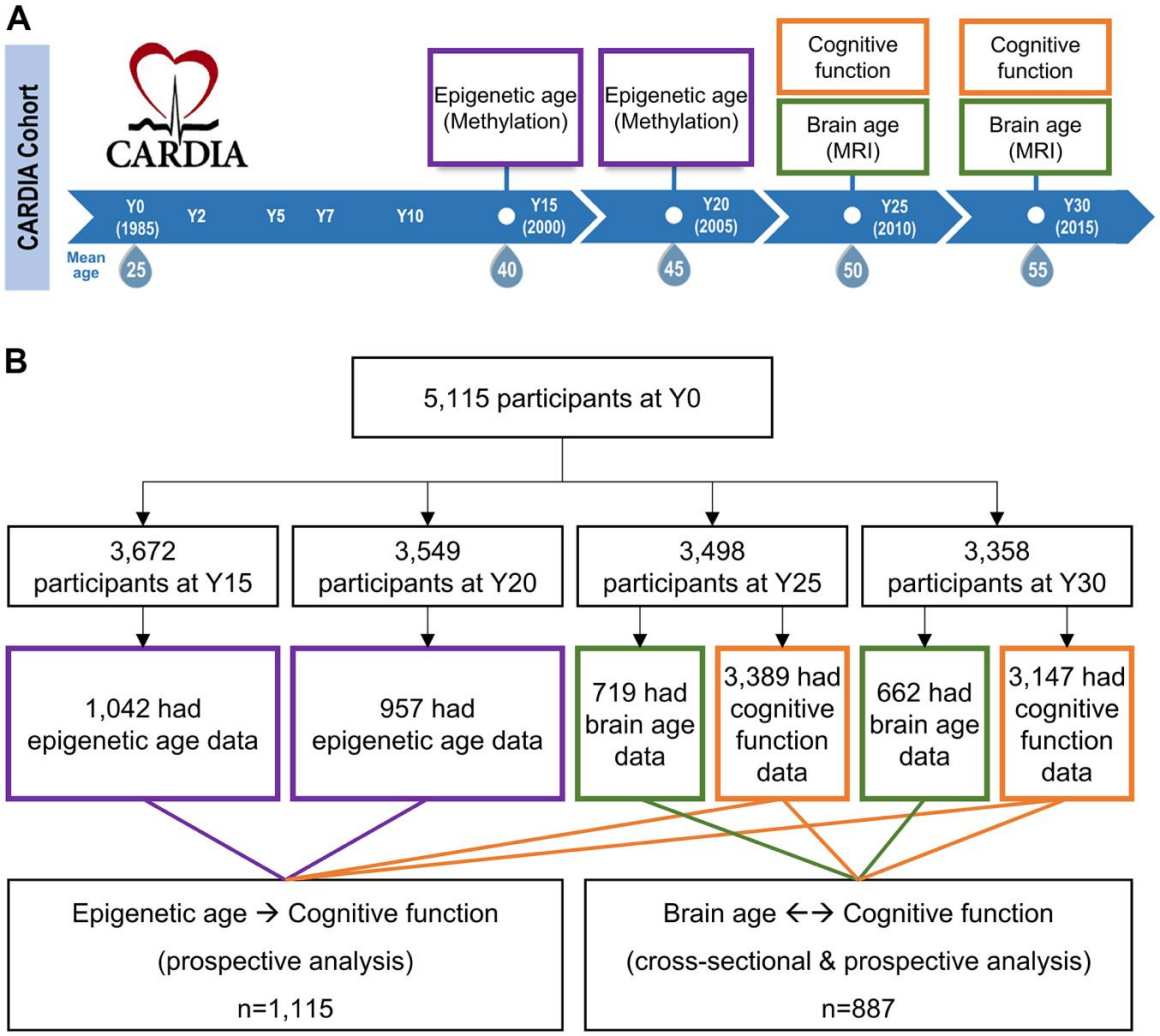
**Study design and eligible study participants.** (**A**) Epigenetic aging data were measured among a randomly selected subset of CARDIA participants at year (Y) Y15 and Y20. Brain aging data were measured at a subset of participants at Y25 and Y30. Cognitive function tests were performed at Y25 and Y30 across almost all CARDIA participants. The DNA methylation was measured at earlier visits before brain MRI because molecular changes could occur years before the brain structural changes. Besides, as a blood-based marker, epigenetic age can be cost-effectively measured at an earlier age. (**B**) Among the 1,042 Y15 and 957 Y20 participants who had methylation data, 881 had methylation data at both visits. Among the 719 Y25 and 662 Y30 participants who had brain MRI data, 488 had MRI data at both visits. About 95% of the CARDIA participants at Y25 and Y30 had cognitive function data. To maximize statistical power, those who had available DNA methylation and cognitive function data were eligible for epigenetic age analysis (a union set of 1,115 participants involved); those who had available brain MRI and cognitive function data were eligible for brain age analysis (a union set of 887 participants involved). There were 326 overlapping participants who had both DNA methylation and brain MRI data.

Epigenetic age, brain age, and cognitive function were measured twice at two consecutive visits (Figure 1). Table 1 shows the overall characteristics of the participants included in our study were representative of the total CARDIA population. The participants had a mean age of 50 and 55 at Y25 and Y30 visits, respectively, with approximately equal representation of both sex and racial groups. The participants included for epigenetic sub-study and brain MRI sub-study were not identical (81% were not overlapped). However, participant characteristics across the epigenetic and brain aging analyses did not significantly differ, indicating comparable study samples between two sub-studies. Significant and moderate correlations were only observed across the epigenetic aging markers (GrimAA, PhenoAA, EEAA, and IEAA, see Methods) but the correlations were weak with SPARE-BAA. (Supplementary Figure 1). Epigenetic age and SPARE-BA were both moderately correlated with chronological age (Pearson’s r=0.37 to 0.55, Supplementary Figure 2).

**Table 1:** Characteristics of study participants at year 25 and Y30.

**Characteristicsmean (SD) / count (%)**	**All CARDIA participants (Y25 and Y30)** **(n = 3,726)**	**Epigenetic age and cognitive function analysis** **(n = 1,115)**	**Brain age and cognitive function analysis** **(n = 887)**	**p-value^1^**
**Age (Y25, year)**	50.2 (3.6)	50.3 (3.5)	50.2 (3.5)	0.396
**Age (Y30, year)**	55.1 (3.6)	55.4 (3.5)	55.2 (3.6)	0.349
**Sex (%)**				
Female	2104 (56.5)	559 (50.1)	466 (52.5)	0.134
Male	1621 (43.5)	556 (49.9)	421 (47.5)	
**Race (N, %)**				
Black	1781 (47.8)	454 (40.7)	368 (41.5)	0.604
White	1945 (52.2)	661 (59.3)	519 (58.5)	
**Education (N, %)**				
High school or less	780 (20.9)	241 (21.6)	193 (21.8)	0.361
Some college	960 (25.8)	297 (26.6)	256 (28.9)	
College graduate or higher	1710 (45.9)	577 (51.7)	438 (49.3)	
**Study field center (N, %)**				
Birmingham	871 (23.4)	269 (24.1)	239 (26.9)	<0.001
Chicago	833 (22.4)	248 (22.2)	-	
Minneapolis	978 (26.2)	288 (25.8)	354 (39.9)	
Oakland	1044 (28.0)	310 (27.9)	294 (33.2)	
**Stroop test (Y25)**	22.8 (10.8)	22.7 (10.9)	22.3 (9.6)	0.373
**Stroop test (Y30)**	23.0 (11.7)	22.5 (11.8)	22.2 (11.4)	0.782
**RAVLT (Long delay recall, Y25)**	8.3 (3.3)	8.4 (3.2)	8.4 (3.3)	0.585
**RAVLT (Long delay recall, Y30)**	8.5 (3.4)	8.7 (3.3)	8.8 (3.4)	0.685
**DSST (Y25)**	69.9 (16.2)	70.6 (16.1)	70.0 (15.6)	0.381
**DSST (Y30)**	67.4 (17.0)	68.5 (16.2)	68.5 (16.3)	0.890

^1^Comparisons between epigenetic age analysis and brain age analysis. P-values were calculated based on t-test for continuous variables and chi-square test for categorical variables. The epigenetic age analysis and brain age analysis involved different yet overlapping CARDIA participants. These overlapping participants (n=326) were removed from the test to ensure the data independence between the comparison groups.

Higher GrimAA was significantly associated with lower subsequent cognitive performance across all three cognitive measures (Table 2). The strength of the associations with cognitive outcomes was evident and similar in both short-term (5-year) and long-term (15-year) prospective analyses, as a result, the 5-year delta association analysis did not yield any significant results, indicating that GrimAA had a persistent, stable prospective association with cognition. IEAA, EEAA, PhenoAA were not associated with any cognitive measures (Supplementary Table 1). For brain aging, higher SPARE-BAA was associated with lower cognitive performance (Table 3). We observed stronger and more significant cross-sectional associations between SPARE-BAA and cognitive function at Y30 (mean age 55) than at Y25 (mean age 50). SPARE-BAA also had prospective associations with cognitive measures 5 years later. Although the 5-year delta analyses did not yield significant association, the directions of associations were consistent with cross-sectional and prospective analyses.

**Table 2:** Association between GrimAA and cognitive function.

**Analysis type**	**Stroop test**				**RAVLT long delay recall**				**DSST**			
	**Coefficient (95% CI)**	**p**	**Adj.p^1^**	**n**	**Coefficient (95% CI)**	**p**	**Adj.p^1^**	**n**	**Coefficient (95% CI)**	**p**	**Adj.p^1^**	**n**
**5-year prospective analysis^2^** (Y20 epigenetic aging vs. Y25 Cognition)	0.194 (0.050,0.338)	0.009	**0.013**	925	-0.046 (-0.087,-0.005)	0.028	**0.028**	932	-0.308 (-0.505,-0.110)	0.002	**0.007**	931
**15-year prospective analysis^2^** (Y15 epigenetic aging vs. Y30 Cognition)	0.231 (0.069,0.394)	0.005	**0.008**	890	-0.048 (-0.091,-0.005)	0.029	**0.029**	905	-0.404 (-0.614,-0.195)	<0.001	**<0.001**	906
**Delta analysis** (5-year change in GrimAA vs. 5-year change in cognition)	0.006 (-0.159, 0.170)	0.946	0.946	741	0.049 (0.000,0.098)	0.049	0.147	754	0.076 (-0.093, 0.245)	0.380	0.569	754

^1^BH-FDR adjustment was applied to account for multiple testing for each aging marker across all the cognitive function tests.

^2^Multiple linear regression models adjusting for age, sex, race, study fields, and education. Beta coefficients indicate changes in cognitive function score by one year greater in GrimAA.

**Table 3:** Association between SPARE-BAA and cognitive function.

**Analysis type**	**Stroop test**				**RAVLT long delay recall**				**DSST**			
	**Coefficient (95% CI)**	**p**	**Adj.p^1^**	**n**	**Coefficient (95% CI)**	**p**	**Adj.p^1^**	**n**	**Coefficient (95% CI)**	**p**	**Adj.p^1^**	**n**
**Cross-sectional analyses^2^** (Y25 SPARE-BAA vs. Y25 Cognition)	0.043 (-0.054, 0.141)	0.382	0.382	704	-0.036 (-0.068,-0.005)	0.024	**0.037**	704	-0.229 (-0.378,-0.080)	0.003	**0.008**	707
**Cross-sectional analyses^2^** (Y30 SPARE- BAA vs. Y30 Cognition)	0.283 (0.158,0.408)	<0.001	**<0.001**	615	-0.042 (-0.078,-0.007)	0.021	**0.021**	620	-0.448 (-0.609,-0.287)	<0.001	**<0.001**	623
**Prospective analyses^2^** (Y25 SPARE- BAA vs. Y30 Cognition)	0.151 (0.022,0.279)	0.022	**0.033**	630	-0.031 (-0.067, 0.004)	0.086	0.086	641	-0.274 (-0.438,-0.111)	0.001	**0.003**	644
**Mixed-effects model^3^**	0.144 (0.059,0.230)	0.001	**0.002**	1319	-0.038 (-0.064,-0.012)	0.004	**0.004**	1324	-0.266 (-0.383,-0.149)	0.000	**<0.001**	1330
**Delta analysis** (5-year change in SPARE-BAA vs. 5-year change in cognition)	0.218 (-0.012, 0.448)	0.064	0.193	466	-0.028 (-0.096, 0.041)	0.430	0.644	472	-0.056 (-0.308, 0.196)	0.663	0.663	472

^1^BH-FDR adjustment was applied to account for multiple testing for each aging marker across all the cognitive function tests.

^2^Multiple linear regression models adjusting for age, sex, race, study fields, and education. Beta coefficients indicate changes in cognitive function score by one year greater in SPARE-BAA.

^3^Mixed-effects model with random intercept incorporating SPARE-BAA at Y25 and Y30, and cognitive function at Y25 and Y30.

We further evaluated the associations between the 5-year changes of age markers over time (Y15 to Y20 for epigenetic age, Y25 to Y30 for brain age) and cognitive function at Y30. Consistent with the 5-year delta association analyses, faster rate of change in SPARE-BA over time, but not any of the epigenetic age markers, was significantly associated with worse performance in the Stroop test (false discovery rate (FDR)=0.007) and DSST (FDR=0.033) (Supplementary Table 2).

Given the significant associations of GrimAA and SPARE-BAA with cognitive function, we further evaluated the effect modifications of sex and carrier status for the *APOE*4 allele. We observed that compared to men, women were more likely to yield stronger associations between GrimAA and cognitive function, especially Stroop test and DSST (Supplementary Table 3), although the interaction tests were not significant after multiple comparison adjustment.

*APOE4* genotype, on the other hand, has been shown to increase the risk of Alzheimer's disease and lowers the age of disease onset [31]. GrimAA was greater than 0 among those who were homozygous for *APOE4* (*APOE* 4/4) and higher than the non-carrier (*APOE* 3/3) and heterozygous (*APOE* 4/3) but not statistically significant (Supplementary Figure 3). SPARE-BAA was roughly equal across three genotype groups. No significant differences were observed after stratifying the association analyses of GrimAA and SPARE-BAA with cognitive function by non-carrier (*APOE* 3/3) vs. carriers (*APOE* 4/3 or 4/4) (Supplementary Table 4).

**Figure 2:**
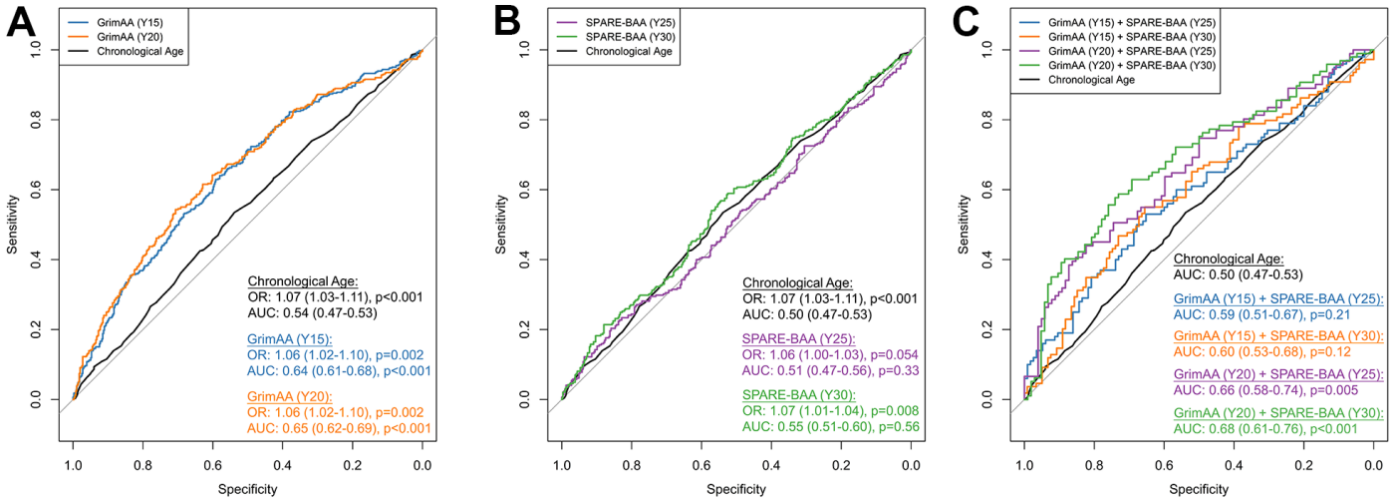
ROC curves of GrimAA (**A**) SPARE-BAA (**B**) and their joint modeling (**C**) in predicting global cognitive status at Y30. The median of the first principal component of Stroop, RAVLT (long delay recall), and DSST test scores (i.e., global cognitive function) measured at Y30 was used to dichotomize the global cognitive status into low (denote by 1) vs. high (denote by 0). The ROC curves were generated using 80/20 training/testing sets with 5-fold cross-validation to avoid overfitting. Associations between two aging markers and Y30 cognitive status evaluated by logistic regression were presented as odds ratio (OR) with every one year greater in GrimAA/SPARE-BAA, adjusting for age, sex, race, study fields, and education. The p-values of AUC were calculated by comparing with the chronological age benchmark AUC curve. GrimAA: GrimAge acceleration; SPARE-BAA: SPARE-BA acceleration; OR: odds ratio; AUC: area under the ROC curve.

Due to the weak correlations between GrimAA and SPARE-BAA (Pearson’s r =0.04-0.18), as well as the strong associations with cognition observed for GrimAA and SPARE-BAA, in particular, we opted to select GrimAA and SPARE-BAA to further explore their additive predictive performance in discriminating future global cognitive status at Y30. Global cognitive status was constructed as a composite score from the first principle components (PC1) of all the three cognitive test scores (see Materials and Methods). PC1 was significantly correlated with all cognitive tests (p < 0.001) with higher PC1 values indicating better cognitive performance (Supplementary Figure 4). As a baseline marker, chronological age showed a significant association with global cognitive status (OR=1.07 per year older, 95%CI: 1.03-1.11, p <0.001) but discrimination was poor (AUC=0.54, 95%CI: 0.47-0.53). Every year greater in Y15 and Y20 GrimAA was associated with 1.06 higher odds of low global cognitive status (95% CI: 1.02-1.10; p=0.002, Figure 2A). SPARE-BAA at Y30 had a more significant association with global cognitive status than Y25 (Figure 2B). GrimAA (AUC = 0.64-0.65) achieved better predictive performance than SPARE-BAA (AUC 0.51-0.55). Although SPARE-BAA alone did not demonstrate discrimination values, combining GrimAA at Y20 and SPARE-BAA at Y30 produced the highest AUC in distinguishing between future high and low global cognition at Y30 (AUC=0.68, 95%CI: 0.61-0.76).

## DISCUSSION

The current study evaluated two classes of new biological aging biomarkers, epigenetic aging and brain aging, as predictors of cognition in a biracial middle-aged population-based cohort. Our results support growing literature indicating that accelerated GrimAge is a robust predictor of adverse health outcomes across both the peripheral and central nervous systems [21, 32, 33]. Additionally, accelerated brain age (as measured via SPARE-BAA) was both cross-sectionally and prospectively associated with lower performance in multiple cognitive domains. GrimAA and SPARE-BAA were not correlated with one another, indicating that they capture distinct facets of biological aging. A combined model with GrimAA and SPARE-BAA improved predictive performance for lower global cognitive status at Y30. Overall, the results indicate that GrimAA and SPARE-BAA are potentially useful indicators of worse cognitive outcomes at midlife- a period in which interventions for preventing irreversible cognitive impairment may be most beneficial [34].

GrimAA was associated with worse cognitive performance across domains and time intervals. Multiple studies have demonstrated GrimAA's relevance as a biological marker for advanced cognitive aging [33, 35, 36]. McCrory et al. examined the associations of IEAA, EEAA, PhenoAA, and GrimAA with performance on cognitive screening tasks in the Irish Longitudinal Study of Ageing, a study of community-dwelling older people in Ireland [32]. Similar to the current results, GrimAA was the only epigenetic aging marker associated with worse cognitive performance. In the Lothian Birth Cohort 1936, GrimAA was found to have significant associations with an array of neurologically-relevant outcomes including worse general cognition, slowed processing speed, and lower perceptual organization scores [33]. Maddock et al. reported that GrimAA predicted decline in verbal memory and processing speed across a 16-year interval [35]. In our study, GrimAA demonstrated largely stable associations with cognitive outcomes over time, highlighting its potential utility for advancing early detection of cognitive vulnerability. Different from the earlier versions of epigenetic age biomarkers, GrimAA is a composite biomarker consisting of blood DNAm surrogates of smoking pack-years as well as seven plasma proteins that are associated with various age-related conditions, including adrenomedullin, β2-microglobulin, cystatin C, growth differentiation factor-15, plasminogen activator inhibitor 1, leptin, and tissue inhibitor metalloproteinase-1 [21]. All of these plasma proteins, together with smoking, have been shown to be associated with cognitive impairment and Alzheimer's disease [37–44]. This is also supported by the significant correlations between the 8 DNAm surrogate components of GrimAA and the 3 cognitive tests in our data (Supplementary Figure 5), which may explain why GrimAA outperformed the other epigenetic age biomarkers in our study.

SPARE-BAA was cross-sectionally and prospectively associated with cognition. In alignment with SPARE-BA’s summarized atrophy pattern with predilection for rostral brain structures, [29] the measure was most strongly associated with tests heavily reliant on frontal lobe function [45, 46]. Cross-sectional analyses demonstrated more robust cognitive associations at Y30 as compared with Y25, suggesting greater sensitivity within older age groups. This observation may help explain the divergence of our results with a prior study that reported null associations between SPARE-BA and cognition when examining a cohort spanning from early adulthood to late-life (20-90 years) [29]. More consistent with the current findings, a study of older adults (50-96 years) reported worse executive function and attentional abilities in the advanced brain aging group with the most significant gray matter atrophy [10]. Future research with broader age ranges is necessary to fully evaluate the potential of time-varying associations of advanced brain aging.

Despite the loss of statistical significance after accounting for multiple comparison, we observed a general trend that women were more likely to yield stronger and more significant associations, particularly for Stroop test and DSST (Supplementary Table 3). This is in line with a recent multi-cohort population study with 34,349 US adults, [47] which suggested that women may experience faster cognitive decline than men- an equivalent to about 5 years faster of cognitive aging. We did not observe significant interactions between *APOE4* and both age acceleration markers. This may be due to the relatively younger population we studied, where the impact of *APOE4* has not yet presented. Evidence has shown that the differences between *APOE4* carriers vs. non-carriers in terms of cognitive ability can become more pronounced with older age [48]. The generally more significant results among the *APOE* non-carrier group we observed may result from more participants (about doubled) than the carrier. Future studies may consider larger sample size when studying this topic among younger population.

GrimAA had a stronger predictive performance for cognition than SPARE-BAA. However, changes in the SPARE-BA and SPARE-BAA index over time showed stronger associations with cognition than the changes in any types of epigenetic age over time, suggesting that it may change more dynamically in temporal association with cognitive decline. Age-related cognitive decline is precipitated by changes in synaptic morphology and function [49]. Structural atrophy develops subsequent to the synaptic changes, [50] which may help explain SPARE-BAA's weaker prognostic significance for cognition in our middle-aged sample. Early changes in synaptic structure and function are associated with alterations in gene expression, which are governed in part by epigenetics [5]. Prior research has indicated both distinct and overlapping epigenetic changes in blood and brain tissue [24]. Consistent with a previous study, [51] blood-derived epigenetic aging may have some, correspondence with epigenetic processes in the brain. Alternatively, as a predictor of multi-organ dysregulation [21], GrimAA may reflect broad homeostatic dysfunction and inflammation capable of inducing adverse outcomes across the peripheral and central nervous systems. GrimAA was weakly correlated with SPARE-BAA, which is consistent with a previous study that blood-based epigenetic age markers may not be well calibrated for measuring biological aging of the brain [52]. Hence, a combined model with both GrimAA and SPARE-BAA had an improved predictive performance for cognitive function, suggesting that they may provide complementary information relevant to accelerated cognitive aging. Despite our ROC curve analyses were robust by using the 80/20 data split and cross-validation, we would like to point out that given the limited sample size of those who had both DNAm data and brain MRI data, this analysis was still exploratory and should be interpreted with caution.

While our study has many strengths, including a large, racially diverse sample and 15 years of longitudinal data collection, the limitations of the study must be considered when interpreting the results. First, epigenetic and brain imaging markers were largely derived from different study participants. While the two subsets of participants had similar demographics and cognitive performance, there may be unmeasured factors that could contribute to bias in the results. Additionally, cognitive function at younger ages was not available, and epigenetic markers were collected at different time points than cognitive and neuroimaging outcomes. Thus, we are unable to evaluate cross-sectional associations with epigenetic aging and cannot directly compare epigenetic and brain aging markers collected at the same time points. Despite this limitation, outcomes with epigenetic markers were generally stable across the timepoints assessed and predictive of cognitive function 10 years later, indicating a long-term marker of aging. Finally, this study in CARDIA had a limited window on the lifespan, and the associations we observed may differ or be better captured at older ages. Future studies conducted in longer follow-up periods will be necessary to evaluate the relative efficacy of epigenetic and brain aging markers for predicting preclinical neurodegenerative disease and incident dementia.

In conclusion, across the four epigenetic aging markers examined, GrimAA was unique in its ability to predict worse cognitive outcomes in our middle-aged CARDIA population. A separate class of biological aging markers derived from neuroimaging outcomes also demonstrated cross-sectional and prospective associations with cognition. Epigenetic aging and brain aging markers may capture distinct facets of cognitive aging. A combined model with epigenetic (GrimAA) and brain (SPARE-BAA) aging markers improved predictive performance for lower cognitive performance. Overall, the results showcase the prognostic significance of biological aging markers for cognitive health. With further validation, epigenetic and brain aging markers may help aid timely identification of individuals at risk for accelerated cognitive decline and promote the development of interventions to preserve optimal functioning across the lifespan.

## MATERIALS AND METHODS

### Study sample

DNA methylation and brain MRI data were generated and re-analyzed in two separate sub-studies in the Coronary Artery Risk Development in Young Adults study (CARDIA), a prospective, multi-center cohort. In 1985-1986, 5,115 self-identified Black and White men and women ages 18-30 were recruited from four urban sites in the US: Birmingham, Chicago, Minneapolis, and Oakland. CARDIA's initial sample was approximately balanced with respect to race, sex, age, education, and study site. CARDIA participants have been followed for over 30 years with high retention rates (>70% of surviving participants attending each in-person examination) [53]. Additional details regarding study design and recruitment, and participant characteristics at baseline, have been reported previously [54]. Our study used data collected from examination Year (Y) 15, Y20, Y25, and Y30 (Figure 1A). DNAm was measured at earlier visits before brain magnetic resonance imaging (MRI) because molecular changes could occur years before the brain structural changes. Besides, as a blood-based marker, epigenetic age can be cost-effectively measured at an earlier age. To maximize statistical power, we utilized all participants (n=1,676) with available data for the corresponding association analyses, which involved 1,115 participants in epigenetic age analysis and 887 participants in brain age analysis (Figure 1B). CARDIA was approved by the institutional review boards at all study sites, and all participants provided written informed consent, including for the collection of DNA from blood.

### DNA sample collection and DNAm profiling

Overnight fasting blood samples were collected in EDTA tubes. DNA was extracted using a PureGene DNA extraction kit (Gentra Systems) and stored at −70° C. Among 3,672 and 3,549 individuals in CARDIA who attended both examinations at Year (Y) 15 and Y20, respectively, we randomly selected 1,200 individuals for DNAm profiling at each examination to achieve a balance sampling within four strata of race and sex from the four CARDIA field centers. Raw DNAm data were preprocessed, QCed, and normalized (Supplementary Methods). Under the stringent QC criteria, we excluded 158 and 243 participants who had low-quality DNA or DNAm data at Y15 and Y20, respectively.

### Epigenetic age acceleration

Epigenetic age estimates were calculated online at https://dnamage.genetics.ucla.edu/new [19]. We generated four epigenetic age estimates at both Y15 and Y20 visits: DNAm GrimAge [21], DNAm PhenoAge [23], Hannum's DNAm Age, [18] and Horvath's DNAm Age [19]. We calculated the corresponding acceleration measures, GrimAge Acceleration (GrimAA), PhenoAge Acceleration (PhenoAA), Extrinsic Epigenetic Age Acceleration (EEAA), and Intrinsic Epigenetic Age Acceleration (IEAA), which are defined as the residuals of a linear model of the corresponding epigenetic age regressed on chronological age and thus independent of chronological age [19].

### Brain MRI measures

Among 3,498 and 3,358 individuals in CARDIA who attended at Y25 and Y30, respectively, 719 at Y25 and 663 at Y30 participated in the CARDIA Brain MRI Sub-study. Exclusion criteria of the MRI Sub-study included contraindication to MRI, possible pregnancy, or a body size that was too large for the MRI tube bore [4, 55]. The participants selected for the CARDIA Brain MRI Sub-study achieved a balance sampling within four strata of race and sex from three of the CARDIA field centers: Birmingham,, Minneapolis, and Oakland. Brain MRI was performed using 3T MR scanners at three CARDIA study field sites (Siemens 3T Tim Trio/VB 15 platform at Oakland and Minneapolis sites, and Philips 3T Achieva/2.6.3.6 platform at Birmingham site). MRI data were transferred to the reading center at the University of Pennsylvania (Section of Biomedical Image Analysis, Department of Radiology) for standardized data processing following quality assurance protocols which have been previously described [55].

### Spatial Patterns of Abnormality for REcognition (SPARE) machine learning-based indices

Among the individuals who underwent brain MRI, we computed the SPARE indices in CARDIA using a previously trained and validated model [28] for all 719 individuals at Y25 and 662 individuals at Y30 (one participant was excluded due to poor MRI data quality) using T1 imaging data and machine learning methods that have been extensively validated in other cohorts [11, 28–30, 56]. Briefly, the SPARE-Brain Age (BA) [10, 29, 56] represents the predicted brain age from a model trained by brain MRI data of cognitively normal individuals from the iSTAGING consortium [56] and harmonized with CARDIA (Supplementary Methods). Similar to DNAm age acceleration, we calculated SPARE-BA acceleration (SPARE-BAA) using the residuals of a linear model of SPARE-BA regressed on chronological age.

### Cognitive tests

A battery of standardized tests to measure cognitive function was first administered in over 90% of the CARDIA participants at the Y25 (n=3,389) and Y30 (n=3,147) visits. This included 1) the Stroop Color and Word Test (Stroop), which evaluates the ability to respond to one stimulus dimension while suppressing the response to another dimension (lower score the better)- an "executive" skill largely attributed to frontal lobe function; [3] 2) the Rey Auditory Verbal Learning Test (RAVLT), which assesses the ability to learn and to recall words (verbal memory) [57]. Results from long delay (10 min) free recall were considered in our analysis with higher scores indicating better performance (range 0-15); and 3) the Digital Symbol Substitution Test (DSST), a subtest of the Wechsler Adult Intelligence Scale (3^rd^ edition) that assesses visual-motor speed, sustained attention, and working memory, [58] with higher scores indicating better performance (range 0-133).

### *APOE* genotyping

APOE genotyping was determined from plasma samples collected at Year 7 examination (1992–1993) by isoelectric focusing and immunoblotting, described previously by Kataoka et al. [59].

### Statistical analysis

All statistical analyses were performed with R (version 4.0.0). Characteristics of participants were compared with Student's t-test for continuous variables and chi-square test for categorical variables. We evaluated the pair-wise correlations between epigenetic aging markers and SPARE-BAA using Pearson's correlation coefficient (*r*). We used multiple linear regression models with epigenetic aging markers or SPARE-BAA treated as independent variables and cognitive tests as dependent variables. For SPARE-BAA, we used mixed-effects model with a random intercept to accounts for the repeated measures of SPARE-BA and cognitive function at Y25 and Y30. For SPARE-BAA, we used mixed-effects model with a random intercept to accounts for the repeated measures of SPARE-BA and cognitive function at both Y25 and Y30. For each marker, p-values of the associations across the cognitive tests were adjusted for multiple testing using Benjamini-Hochberg False Discovery Rate (FDR) [60]. All models were adjusted for age, sex, race, study site, and education as covariates. We constructed a composite global cognitive function score using the first principal component (PC1) [61] across the Y30 cognitive tests: Stroop test, RAVLT long delay recall, and DSST. We dichotomized the PC1 using its median to define high (coded as 0) vs. low (coded as 1) global cognitive status. Global cognitive status at Y30 was predicted with logistic regression and tested the odds ratio (OR) using biological aging markers, i.e., epigenetic age acceleration markers at Y15/Y20 and SPARE brain metrics at Y25/Y30, adjusting for the covariates mentioned above. To evaluate the additive predictive performance of both epigenetic age acceleration and SPARE brain metrics, we performed receiver operating characteristic (ROC) curve analysis by modeling both aging markers together. All ROC results were performed based on a 5-fold cross-validation (i.e., 80/20 ratio training/testing dataset split) to avoid overfitting. We used R package pROC to estimate 95% confidence intervals (CI) of the area under the ROC curves (AUC) with DeLong test and to compare AUC between two curves with bootstrap test [62].

## Supplementary Material

Supplementary Methods

Supplementary Figures

Supplementary Tables
