# Supplementary material for: Mid-life epigenetic age, neuroimaging brain age, and cognitive function: coronary artery risk development in young adults (CARDIA) study
Source: Aging (Albany NY). 2022 Feb 27;14(4):1691–712. doi: 10.18632/aging.203918 (PMC8908939; doi:10.18632/aging.203918)
Supplement: Supplementary Methods [file aging-14-203918-s001.pdf]

SUPPLEMENTARY METHODS

Illumina EPIC array methylation data quality control and preprocessing

Infinium MethylationEPIC BeadChip raw data (IDAT files) were generated. The R package ENmix [1] was used for quality control with default parameter settings. Low-quality methylation measurements were identified by detection p-value $<10^{-6}$ or the number of beads <3 [1]. We excluded 6,209 CpGs with a detection rate <95% and 87 samples with a percentage of low-quality methylation measurements >5% or extremely low intensity of bisulfite conversion probes [1]. We further removed 95 samples that were extreme outliers, as defined by Tukey's method [i.e., <25th percentile – 3 * interquartile range (IQR) or >75th percentile + 3 * IQR] [2] and based on the average total intensity value [intensity of the unmethylated signal (U) + intensity of the methylated signal (M)] or β value [M / (U + M + 100)] across CpG probes. The remaining samples were preprocessed using *preprocessIllumina* function in minfi package [3] before the estimations of epigenetic age.

Spatial patterns of abnormality for recognition (SPARE) machine learning-based indices

The SPARE-BA method relies on a multivariate pattern regression model to predict individualized brain age for each participant, similar to our previous work [4, 5]. Support vector regression model (radial basis function kernel) was trained with the T1-MR scans using regional volumetric measures for structures. The training set included only cognitively normal subjects. The training set for SPARE-BA consisted of (n=8,284) subjects from the iSTAGING consortium [6].

REFERENCES

1. Xu Z, Niu L, Li L, Taylor JA. ENmix: a novel background correction method for Illumina HumanMethylation450 BeadChip. Nucleic Acids Res. 2016; 44:e20. https://doi.org/10.1093/nar/gkv907 PMID:26384415
2. Tukey J. Exploratory Data Analysis: Pearson. 1977.
3. Aryee MJ, Jaffe AE, Corrada-Bravo H, Ladd-Acosta C, Feinberg AP, Hansen KD, Irizarry RA. Minfi: a flexible and comprehensive Bioconductor package for the analysis of Infinium DNA methylation microarrays. Bioinformatics. 2014; 30:1363–9. https://doi.org/10.1093/bioinformatics/btu049 PMID:24478339
4. Habes M, Erus G, Toledo JB, Zhang T, Bryan N, Launer LJ, Rosseel Y, Janowitz D, Doshi J, Van der Auwera S, von Sarnowski B, Hegenscheid K, Hosten N, et al. White matter hyperintensities and imaging patterns of brain ageing in the general population. Brain. 2016; 139:1164–79. https://doi.org/10.1093/brain/aww008 PMID:26912649
5. Eavani H, Habes M, Satterthwaite TD, An Y, Hsieh MK, Honnorat N, Erus G, Doshi J, Ferrucci L, Beason-Held LL, Resnick SM, Davatzikos C. Heterogeneity of structural and functional imaging patterns of advanced brain aging revealed via machine learning methods. Neurobiol Aging. 2018; 71:41–50. https://doi.org/10.1016/j.neurobiolaging.2018.06.013 PMID:30077821
6. Habes M, Pomponio R, Shou H, Doshi J, Mamourian E, Erus G, Nasrallah I, Launer LJ, Rashid T, Bilgel M, Fan Y, Toledo JB, Yaffe K, et al. The Brain Chart of Aging: Machine-learning analytics reveals links between brain aging, white matter disease, amyloid burden, and cognition in the iSTAGING consortium of 10,216 harmonized MR scans. Alzheimers Dement. 2021; 17:89–102. https://doi.org/10.1002/alz.12178 PMID:32920988
