# Supplementary material for: Mid-life epigenetic age, neuroimaging brain age, and cognitive function: coronary artery risk development in young adults (CARDIA) study
Source: Aging (Albany NY). 2022 Feb 27;14(4):1691–712. doi: 10.18632/aging.203918 (PMC8908939; doi:10.18632/aging.203918)
Supplement: Supplementary Figures [file aging-14-203918-s002.pdf]

SUPPLEMENTARY FIGURES

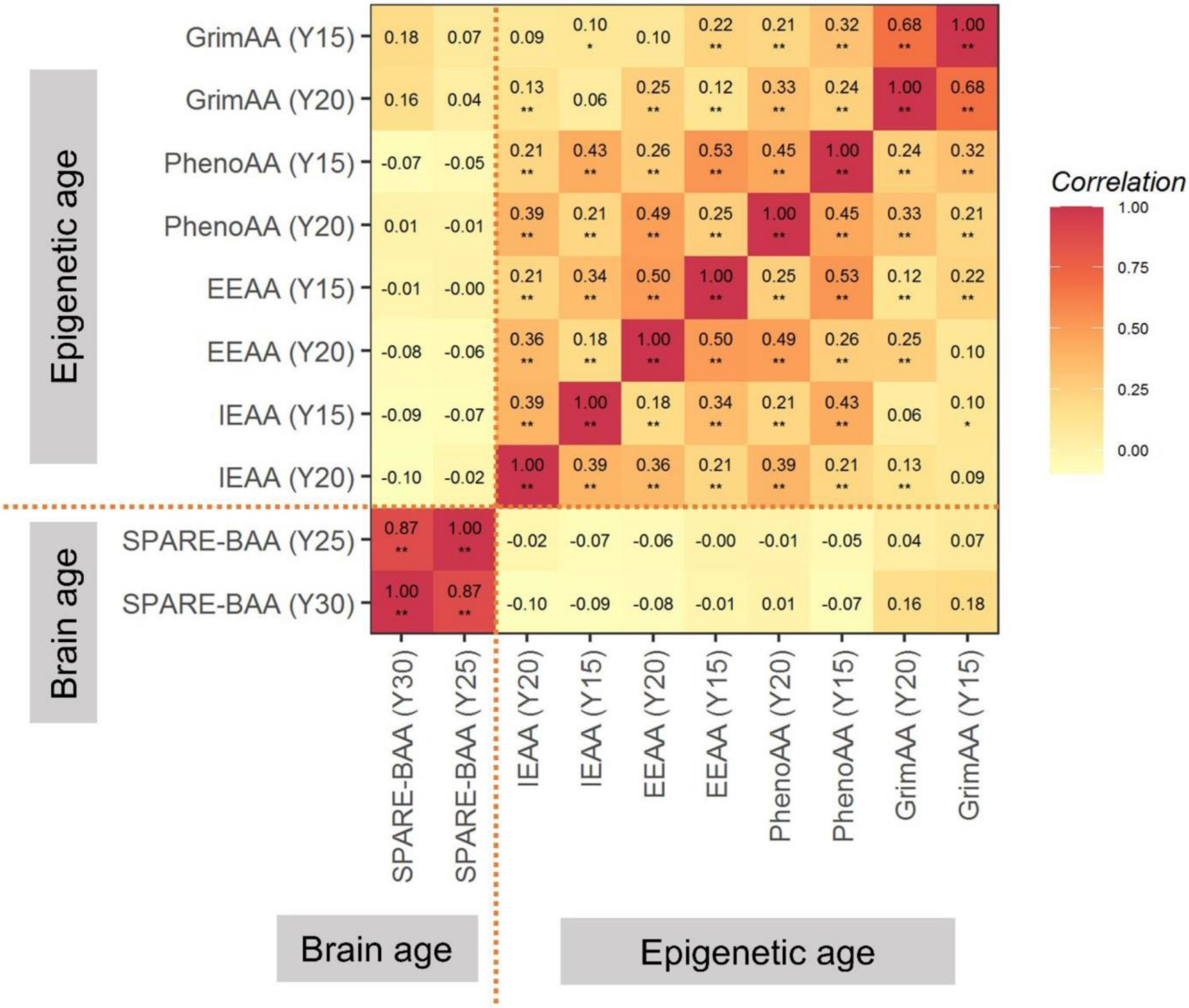

Supplementary Figure 1. Pairwise correlations of epigenetic aging markers and brain aging markers. Multiple comparisons were adjusted using the Holm-Bonferroni method. *: adjusted-p <0.05; **: adjusted-p <0.01.

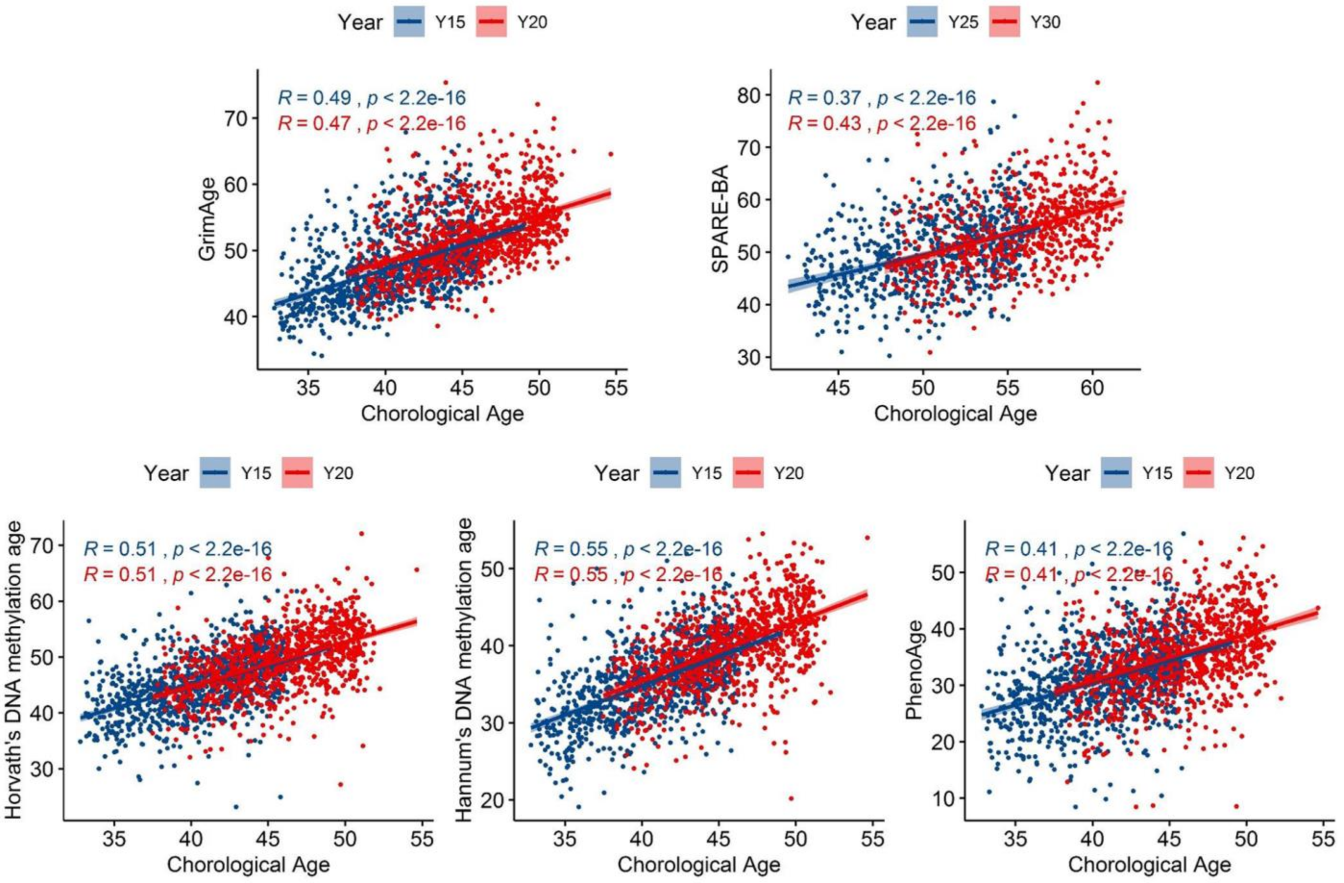

Supplementary Figure 2. Correlations between epigenetic age, brain age, and chronological age.

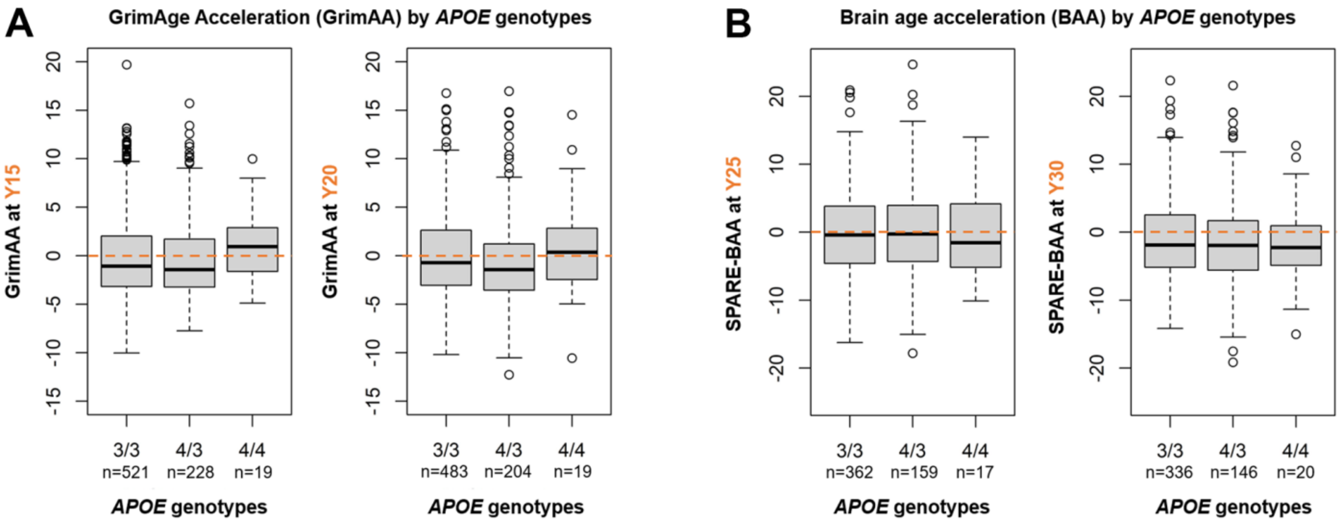

Supplementary Figure 3. GrimAge acceleration and SPARE brain age acceleration by *APOE* genotypes. (**A**) Y15 and Y20 GrimAA by APOE genotypes. (**B**) Y25 and Y30 SPARE-BAA by *APOE* genotypes. GrimAA was greater than 0 among those who were homozygous carrier (*APOE* 4/4) and higher than the non-carrier (*APOE* 3/3) and heterozygous (*APOE* 4/3) but not statistically significant (p <0.05). SPARE-BAA was roughly equal across three genotype groups.

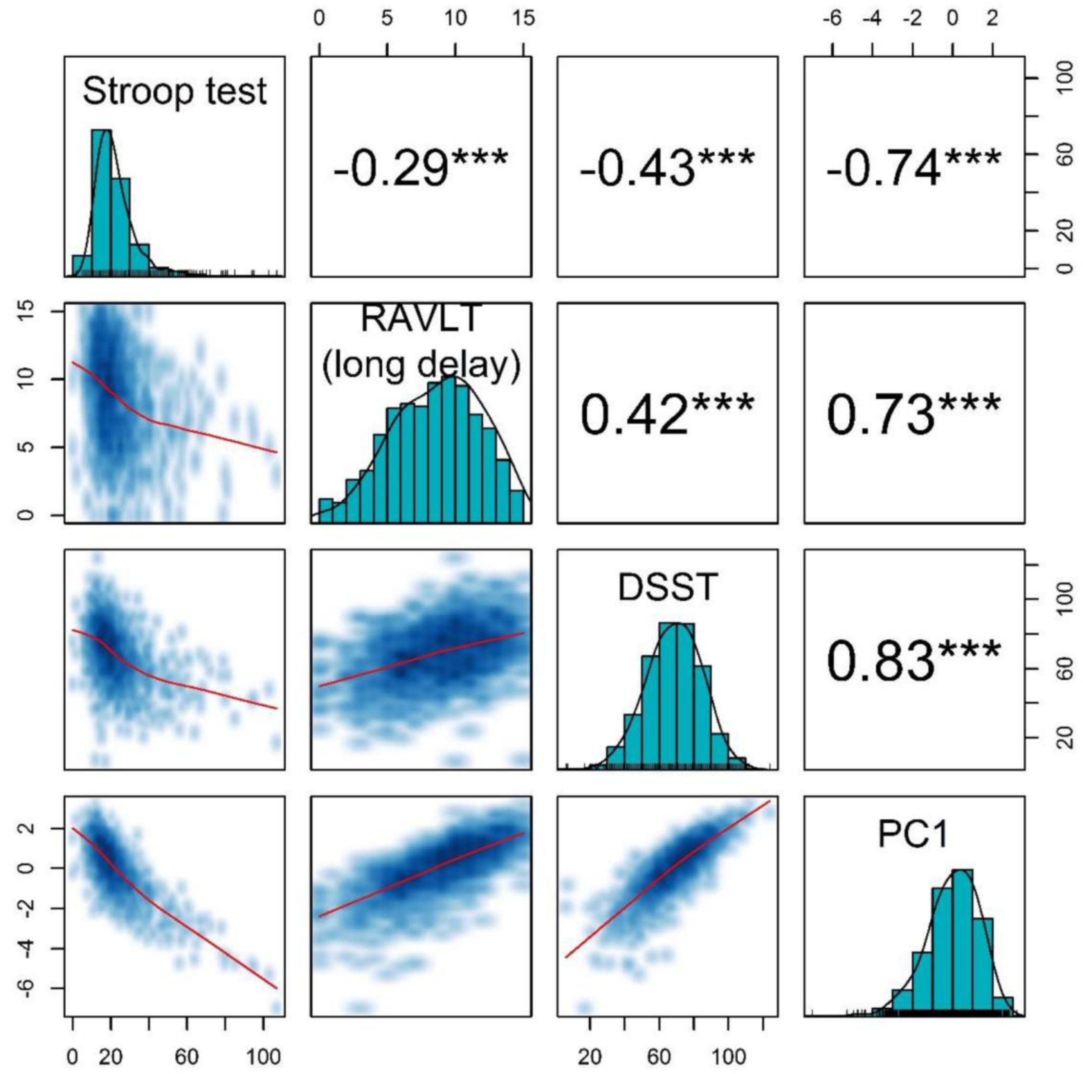

Supplementary Figure 4. Correlations between cognitive testing and the first principal component (PC1) across the tests. The PC1 was negatively associated with Stroop test and positively correlated with RAVLT long delay free recall and DSST. Higher PC1 levels indicate better cognitive performance. The star symbols beside the Pearson's correlation indicate the significance of the correlation test.

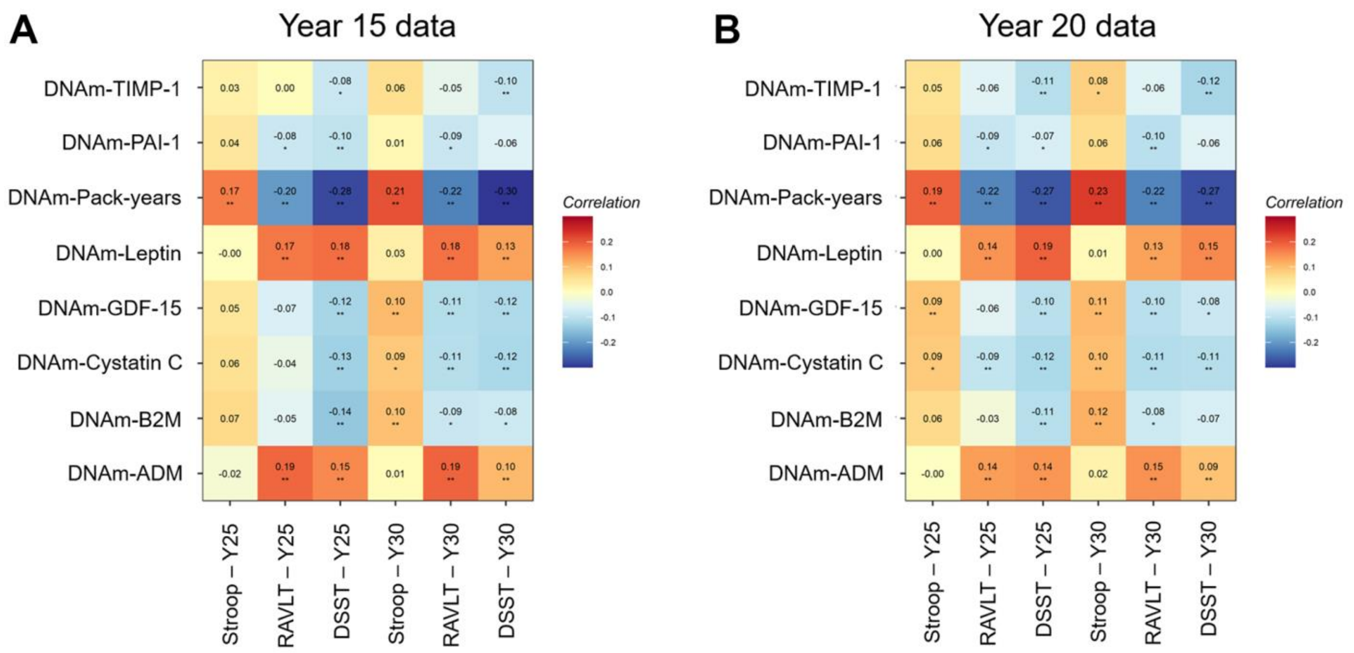

Supplementary Figure 5. Correlation heatmaps between DNA methylation surrogates components of GrimAge and cognitive testings. (**A**) GrimAge methylation components were estimated using Y15 methylation data. (**B**) GrimAge methylation components were estimated using Y20 methylation data. All seven DNA methylation surrogates of plasma protein and pack years of GrimAge were significantly correlated with at least one type of the 3 cognitive testings. In general, Y30 cognition measures resulted in higher correlation compared to Y25 cognition measures. DNAm: DNA methylation; ADM: adrenomedullin; B2M: β2-microglobulin; GDF-15: growth differentiation factor-15; PAI-1: plasminogen activator inhibitor 1; TIMP-1: tissue inhibitor metalloproteinase-1.
