# Supplementary material for: Mid-life epigenetic age, neuroimaging brain age, and cognitive function: coronary artery risk development in young adults (CARDIA) study
Source: Aging (Albany NY). 2022 Feb 27;14(4):1691–712. doi: 10.18632/aging.203918 (PMC8908939; doi:10.18632/aging.203918)
Supplement: Supplementary Tables [file aging-14-203918-s003.pdf]

SUPPLEMENTARY TABLES

Supplementary Table 1. Associations between IEAA, EEAA, and PhenoAA vs. cognitive function.

Analysis type	Epigenetic aging marker	Stroop test				RAVLT-long delay free recall				DSST			
		Coefficient (95% CI)	p	Adj.p[1]	n	Coefficient (95% CI)	p	Adj.p[1]	n	Coefficient (95% CI)	p	Adj.p[1]	n
**5-year prospective analysis[2]** (Y20 epigenetic aging vs. Y25 Cognition)	**IEAA**	0.027 (-0.112, 0.166)	0.701	0.943	920	0.001 (-0.038, 0.041)	0.943	0.943	927	0.066 (-0.126, 0.259)	0.498	0.943	926
	**EEAA**	0.060 (-0.067, 0.186)	0.357	0.453	923	-0.014 (-0.049, 0.022)	0.453	0.453	930	-0.140 (-0.315, 0.034)	0.115	0.345	929
	**PhenoAA**	-0.013 (-0.115, 0.088)	0.795	0.795	924	-0.018 (-0.046, 0.011)	0.220	0.330	931	-0.157 (-0.297,-0.018)	0.027	0.082	930
**15-year prospective analysis[2]** (Y15 epigenetic aging vs. Y30 Cognition)	**IEAA**	0.031 (-0.128, 0.191)	0.700	0.700	888	0.025 (-0.017, 0.067)	0.248	0.470	903	-0.107 (-0.314, 0.101)	0.313	0.470	904
	**EEAA**	0.006 (-0.129, 0.142)	0.926	0.926	890	0.005 (-0.031, 0.041)	0.795	0.926	905	-0.094 (-0.269, 0.082)	0.294	0.883	906
	**PhenoAA**	0.062 (-0.054, 0.178)	0.298	0.437	890	-0.012 (-0.043, 0.019)	0.437	0.437	905	-0.087 (-0.237, 0.062)	0.253	0.437	906

[1]BH-FDR adjustment was applied to account for multiple testing for each aging marker across all the cognitive function tests.
[2]Multiple linear regression models adjusting for age, sex, race, study fields, and education.

Supplementary Table 2. Associations between a five-year change of epigenetic age (Y15-Y20) and brain age (Y25-Y30) vs. cognitive function at Y30.

Aging marker	Stroop test (Y30)				RAVLT-long delay free recall (Y30)				DSST (Y30)			
	Coefficient (95% CI)	p	Adj.p[1]	n	Coefficient (95% CI)	p	Adj.p[1]	n	Coefficient (95% CI)	p	Adj.p[1]	n
Horvath's epigenetic age (5-year change)	0.097 (-0.041, 0.235)	0.169	0.169	757	0.028 (-0.011, 0.066)	0.161	0.169	768	0.162 (-0.028, 0.352)	0.094	0.169	770
Hannum's epigenetic age (5-year change)	0.098 (-0.071, 0.266)	0.255	0.395	758	0.026 (-0.021, 0.073)	0.273	0.395	769	0.100 (-0.130, 0.329)	0.395	0.395	771
PhenoAge (5-year change)	0.008 (-0.100, 0.116)	0.887	0.887	759	0.003 (-0.027, 0.033)	0.832	0.887	770	-0.060 (-0.208, 0.088)	0.429	0.887	772
GrimAge (5-year change)	0.106 (-0.084, 0.296)	0.276	0.423	759	0.006 (-0.047, 0.058)	0.836	0.836	770	0.144 (-0.118, 0.406)	0.282	0.423	772
SPARE-BA (5-year change)	0.478 (0.186,0.770)	0.001	**0.004**	469	-0.017 (-0.101, 0.068)	0.701	0.701	474	-0.454 (-0.843,-0.066)	0.022	**0.033**	475

Note: Since the changes of the aging marker over time were evaluated, the age estimates rather than the age acceleration were used.
[1]BH-FDR adjustment was applied to account for multiple testing for each aging marker across all the cognitive function tests.
[2]Multiple linear regression models adjusting for age, sex, race, study fields, and education.

Supplementary Table 3. Associations between GrimAge acceleration and SPARE-brain age acceleration vs. cognitive function by sex.

Analysis type		Stroop test				RAVLT long delay recall				DSST			
		**Coefficient (95% CI)**	**p**	**Adj.p[1]**	**n**	**Coefficient (95% CI)**	**p**	**Adj.p[1]**	**n**	**Coefficient (95% CI)**	**p**	**Adj.p[1]**	**n**
**5-year prospective analysis[2]** (Y20 epigenetic aging vs.Y25 Cognition)	**Women**	0.187 (-0.011, 0.384)	0.065	0.097	470	-0.036 (-0.092, 0.019)	0.201	0.201	473	-0.341 (-0.623,-0.059)	0.018	0.055	474
	**Men**	0.196 (-0.016, 0.407)	0.070	0.071	455	-0.056 (-0.116, 0.005)	0.071	0.071	459	-0.292 (-0.569,-0.015)	0.039	0.071	457
	Interaction Adj.p[1]	0.808				0.808				0.808			
**15-year prospective analysis[2]** (Y15 epigenetic aging vs. Y30 Cognition)	**Women**	0.427 (0.204,0.651)	<0.001	**<0.001**	461	-0.051 (-0.110, 0.007)	0.087	0.087	462	-0.608 (-0.928,-0.288)	<0.001	**<0.001**	466
	**Men**	0.042 (-0.195, 0.279)	0.731	0.731	429	-0.043 (-0.106, 0.021)	0.189	0.284	443	-0.235 (-0.505, 0.036)	0.090	0.269	440
	Interaction Adj.p[1]	**0.056**				0.723				**0.091**			
**Cross-sectional analyses[2]** (Y25 SPARE-BAA vs. Y25 Cognition)	**Women**	0.154 (0.025,0.284)	0.020	**0.030**	367	-0.036 (-0.078, 0.006)	0.098	0.098	366	-0.258 (-0.470,-0.046)	0.017	**0.030**	369
	**Men**	-0.050 (-0.196, 0.096)	0.505	0.505	337	-0.042 (-0.090, 0.005)	0.081	0.122	338	-0.234 (-0.441,-0.027)	0.027	0.082	338
	Interaction Adj.p[1]	0.108				0.820				0.820			
**Cross-sectional analyses[2]** (Y30 SPARE- BAA vs. Y30 Cognition)	**Women**	0.158 (0.016,0.299)	0.029	**0.044**	324	-0.019 (-0.065, 0.027)	0.411	0.411	322	-0.323 (-0.565,-0.081)	0.009	**0.028**	324
	**Men**	0.424 (0.214,0.633)	<0.001	**<0.001**	291	-0.072 (-0.127,-0.016)	0.012	0.012	298	-0.610 (-0.819,-0.400)	<0.001	**<0.001**	299
	Interaction Adj.p[1]	0.105				0.139				0.105			
**Prospective analyses[2]** (Y25 SPARE- BAA vs. Y30 Cognition)	**Women**	0.176 (0.027,0.326)	0.022	**0.032**	345	-0.038 (-0.085, 0.008)	0.107	0.107	344	-0.317 (-0.556,-0.079)	0.010	**0.029**	346
	**Men**	0.141 (-0.076, 0.359)	0.203	0.305	285	-0.027 (-0.083, 0.029)	0.346	0.346	297	-0.273 (-0.493,-0.052)	0.016	0.049	298
	Interaction Adj.p[1]	0.960				0.960				0.960			

[1]BH-FDR adjustment was applied to account for multiple testing for each aging marker across all the cognitive function tests.
[2]Multiple linear regression models adjusting for age, race, study fields, and education.

Supplementary Table 4. Associations between GrimAge acceleration and SPARE-brain age acceleration vs. cognitive function by *APOE* genotype (*APOE* 3/3 non-carrier vs. *APOE* 4/3 or 4/4 carrier).

Analysis type		Stroop test				RAVLT long delay recall				DSST			
		**Coefficient (95% CI)**	**p**	**Adj.p[1]**	**n**	**Coefficient (95% CI)**	**p**	**Adj.p[1]**	**n**	**Coefficient (95% CI)**	**p**	**Adj.p[1]**	**n**
**5-year prospective analysis[2]** (Y20 epigenetic aging vs. Y25 Cognition)	***APOE* 3/3 (Non-carrier)**	0.280 (0.079,0.482)	0.007	**0.019**	470	-0.053 (-0.112, 0.006)	0.081	0.081	474	-0.364 (-0.650,-0.078)	0.013	**0.019**	470
	***APOE* 4/3 or 4/4 (Carrier)**	0.055 (-0.239, 0.348)	0.716	0.957	216	-0.002 (-0.084, 0.080)	0.957	0.957	216	-0.260 (-0.654, 0.135)	0.198	0.595	217
	Interaction Adj.p[1]	0.248				0.390				0.784			
**15-year prospective analysis[2]** (Y15 epigenetic aging vs. Y30 Cognition)	***APOE* 3/3 (Non-carrier)**	0.224 (0.028,0.419)	0.025	**0.038**	451	-0.013 (-0.075, 0.049)	0.672	0.672	455	-0.363 (-0.665,-0.060)	0.019	**0.038**	455
	***APOE* 4/3 or 4/4 (Carrier)**	-0.039 (-0.407, 0.329)	0.836	0.836	217	-0.054 (-0.138, 0.030)	0.211	0.316	221	-0.445 (-0.860,-0.030)	0.037	0.111	221
	Interaction Adj.p[1]	0.374				0.833				0.833			
**Cross-sectional analyses[2]** (Y25 SPARE-BAA vs. Y25 Cognition)	***APOE* 3/3 (Non-carrier)**	-0.066 (-0.186, 0.055)	0.285	0.285	358	-0.032 (-0.078, 0.014)	0.176	0.263	358	-0.185 (-0.409, 0.039)	0.107	0.263	359
	***APOE* 4/3 or 4/4 (Carrier)**	0.164 (-0.036, 0.363)	0.110	0.165	174	-0.042 (-0.104, 0.019)	0.179	0.179	174	-0.362 (-0.657,-0.068)	0.017	0.051	174
	Interaction Adj.p[1]	0.050				0.636				0.560			
**Cross-sectional analyses[2]** (Y30 SPARE- BAA vs. Y30 Cognition)	***APOE* 3/3 (Non-carrier)**	0.319 (0.146,0.493)	<0.001	**0.001**	318	-0.019 (-0.073, 0.036)	0.502	0.502	318	-0.514 (-0.767,-0.261)	<0.001	**<0.001**	319
	***APOE* 4/3 or 4/4 (Carrier)**	0.370 (0.133,0.607)	0.003	**0.003**	160	-0.108 (-0.169,-0.048)	0.001	**0.001**	161	-0.582 (-0.836,-0.329)	<0.001	**<0.001**	163
	Interaction Adj.p[1]	0.721				0.118				0.721			
**Prospective analyses[2]** (Y25 SPARE- BAA vs. Y30 Cognition)	***APOE* 3/3 (Non-carrier)**	0.167 (0.000,0.333)	0.051	0.076	323	-0.020 (-0.070, 0.031)	0.449	0.449	328	-0.312 (-0.546,-0.078)	0.010	**0.029**	329
	***APOE* 4/3 or 4/4 (Carrier)**	0.207 (-0.048, 0.462)	0.113	0.113	156	-0.066 (-0.140, 0.008)	0.082	0.113	157	-0.283 (-0.622, 0.055)	0.103	0.113	158
	Interaction Adj.p[1]	0.839				0.732				0.883			

[1]BH-FDR adjustment was applied to account for multiple testing for each aging marker across all the cognitive function tests.
[2]Multiple linear regression models adjusting for age, sex, race, study fields, and education.
